# Comparison of Fatal Injuries Resulting from Tractor and High Speed Motorcycle Accidents in Turkey: A Multicenter Study

**DOI:** 10.1155/2019/9471407

**Published:** 2019-05-02

**Authors:** Suna Eraybar, Serhat Atmaca, Yasemin Nennicioglu, Gokhan Torun, Okan Aydin, Behçet Varisli, Nuran Sandal, Tunç Buyukyilmaz, Murat Seyit, Harun Yildirim, Erol Armagan

**Affiliations:** ^1^Yuksek Ihtisas Education and Research Hospital, Emergency Department, Mimar Sinan Mah. Emniyet Cad. Polis Okulu Karşısı Yıldırım, Bursa, Turkey; ^2^Uludag University, Faculty of Medicine, Emergency Department, Bursa, Turkey; ^3^Canakkale State Hospital, Emergency Service, Canakkale, Turkey; ^4^Inegol State Hospital, Emergency Service, İnegol, Bursa, Turkey; ^5^Balıkesir Ataturk State Hospital, Emergency Service, Balıkesir, Turkey; ^6^Kutahya Evliya Çelebi Education and Research Hospital, Emergency Department, Kutahya, Turkey

## Abstract

**Aim:**

Injuries are among the main causes of mortality and morbidity all over the world, and effective initial triage of these patients can determine the thin line between death and life. Tractor accidents and related injuries are significant problems particularly in rural areas. However, major trauma classification systems do not include tractor accidents as a criterion for trauma team activation or transportation of the patients to a trauma center. This study evaluated the general characteristics and outcomes of tractor accidents in comparison to motorcycle accidents, which are considered as a comparison criterion for major trauma.

**Materials and Methods:**

This is a multicenter study conducted in 6 emergency departments in 4 cities over a six month period. All cases over 18 years of age who were admitted to emergency service due to tractor or motorcycle accidents and meet the criteria were included in the study. The general characteristics and outcomes of both trauma types were compared to determine whether tractor accident should be considered as major traumas.

**Results:**

Eighty-eight patients had a tractor accident, and 339 patients had a motorcycle accident. The tractor accident victims were significantly younger (p<0.001), and the proportion of females was higher in this group (p=0.001). Glasgow coma score (p=0.062), revised trauma score (p=0.201), duration from incident to admission (p=0.481), and route of admission (p=0.810) were similar between both accident types. The rates of thoracic traumas (42% versus 23%, p<0.001) and spinal injuries (17% versus 5.9%, p=0.002) were significantly higher in tractor accidents. The hospitalization rates of the patients were significantly higher in tractor accidents (p=0.008).

**Conclusion:**

The findings of this study support the hypothesis that tractor accidents should be included in the criteria of ATLS major trauma classification system and trauma team activation procedures.

## 1. Introduction

Injuries, particularly due to traffic accidents, are among the main causes of mortality and morbidity across the world [1]. The World Health Organization (WHO) reported that mortality due to road traffic accidents is higher in South-Western Asia than the other parts of the world. According to the data from the Turkish Statistics Institute (TSI), a total of 1.3 million traffic accidents occurred in 2015 in Turkey, and 14% of them involved death and personal injury [2]. By these figures, Turkey has also a significant burden of injuries related to motor vehicle accidents. Among these accidents, tractor accidents are the most frequent causes of mortality in agricultural workers and rollover of the tractors is the primary mechanism that causes death in more than 50% of those accidents [3, 4]. Tractor accidents are not only related to agricultural work but also related to transportation in rural parts of the country. According to the joint report of TSI and Security General Directorate, 1.1% of all accidents involving death and personal injury in 2015 were related to tractor accidents [5].

The management of an injured patient is one of the mainstays of emergency medicine and emergency teams play a crucial role in the management of these patients [6]. The general approach to a trauma patient in an emergency department begins with an efficient triage. Such patients are evaluated according to the American College of Surgeons' Advanced Trauma Life Support (ATLS) criteria at our emergency departments, and if a trauma team is unavailable, then consultations with relevant specialties are coordinated according to these criteria. The activation of trauma teams in different centers may depend on different criteria; however, generally the backbone of those criteria depends to recommendations from the American College of Surgeons' Committee on Trauma [7, 8]. The criteria applied in our trauma centers include abnormal physiological findings, anatomical injuries, mechanism of injuries, and accompanying conditions, and if one of these criteria is present then the trauma team is activated. The mechanisms of injuries include various etiologies of motor vehicle accidents, and motorcycle accidents are one of those criteria. Although the incidence of tractor accidents is not as high as motorcycle accidents, the consequences are comparable; however, traumatic injuries due to tractor accidents are not included in the criteria for trauma team activation systems.

Since agricultural work is one of the most common occupations all over the world as well as in our country, trauma due to tractor accidents creates a significant burden on emergency health services, particularly in rural areas. The aim of this study is to evaluate the general characteristics of tractor accidents by comparing them with motorcycle accidents and assess whether they should be included among the major trauma criteria.

## 2. Materials and Methods

This study was conducted in 6 emergency departments (Yuksek Ihtisas Education and Research Hospital as the coordinating center, 2 university hospitals, 3 state hospitals in city centers, and 1 state hospital in a district) in 4 cities (Bursa, Balikesir, Canakkale, and Kutahya). Ethical committee approval was obtained by local ethical committee of Yuksek Ihtisas Education and Research Hospital during the study planning phase. All cases over 18 years of age admitted to emergency service due to a tractor accident were included in the study. The comparison group was formed of individuals that had a motorcycle accident, which was accounted as a major trauma criterion according to the mechanism of the accident. Patients below 18 year of age and patients who could not provide consent to participate to the study by oneself or by a relative were not included in the study population. Only data from hospital records of patients were used in the analyses, and no additional evaluations, tests, or imaging studies were performed.

A case report form was prepared for tractor accidents, which included the type of transportation to emergency department, the chief of the emergency transport team (doctor, paramedic, and emergency medical technician), trauma team activation criteria, patient characteristics (age, sex, and comorbidities), ATLS criteria, physical examination findings, injuries, laboratory and imaging studies, hospitalization and operational interventions, and duration of stay in emergency room.

The same data was also collected for the motorcycle accident victims for comparisons between the study and control groups. The motorcycle accidents were considered as high-kinetic energy traumas, and tractor accidents were considered as low-kinetic energy traumas. The data collected for both of these groups were compared to obtain a solid understanding of the differences between these groups.

### 2.1. Statistical Analyses

Descriptive analyses of variables were presented as mean, standard deviation, median, minimum, and maximum values, frequency, and percent. Normal distribution was tested by Kolmogorov-Smirnov test. Quantitative and qualitative data were compared with Mann-Whitney U test and Chi-square test, respectively. p <0.05 was considered statistically significant. All analyses were performed with SPSS 22.0 software.

## 3. Results

This study included a total of 427 patients admitted to emergency service with a trauma due to either a tractor (n=88) or a motorcycle (n=339) accident. The general characteristics of patients are presented in Table 1. Rollover of the tractors was the primary mechanism in %50 cases (n: 44); falls and run over rates were %30, 7 (n: 27) and %19, 3 (n: 17), respectively. The tractor accident victims were significantly younger (p<0.001), and proportion of females was higher in this group (p=0.001). However, the Glasgow coma score (GCS) (p=0.062), revised trauma score (RTS) (p=0.201), duration from incident to admission (p=0.481), and route of admission (p=0.810) were similar between both accident types. The types of traumas in both accident types are shown in Table 2. The rates of thoracic traumas were significantly higher in tractor accidents (42% versus 23%, p<0.001). Similarly, spinal injuries were also higher in tractor accidents (17% versus 5.9%, p=0.002). Rates of head trauma (p=0.125) and maxillofacial injury (p=0.864) were similar between groups. Although abdominal trauma (p=0.053) and pelvic injury (p=0.062) rates were higher in tractor accidents, the differences were not statistically significant between groups.

The patients had undergone computerized tomography examinations if they had major trauma in different body sites.  Table 3 summarizes the findings in CT according to the site. The evaluations revealed that cranial (p=0.230) and facial CT (p=0.266) findings were similar between two types of accidents. However, more positive findings were identified in thoracic CT (p<0.001), abdominal CT (p=0.011), pelvic CT (p=0.050), and spinal CT (p<0.001) in tractor accidents. And for the motorcycle accidents, more positive findings were identified in extremity CT evaluations.

The patient outcomes are presented in Table 4. The hospitalization rates of the patients were significantly higher in tractor accidents (p=0.008). Thirty-three percent of patients were hospitalized to inpatient clinics, and 15.9% were hospitalized in intensive care unit. These rates were 22.1% and 9.1% in motorcycle accidents, respectively. Victims of tractor accidents were primarily hospitalized in neurosurgery (11.4%) and orthopedics (10.2%) departments, and motorcycle accident victims were primarily hospitalized in orthopedics department (12.7%).

## 4. Discussion

This study evaluated the general characteristics of patients admitted to emergency services with a diagnosis of tractor accidents and compared the demographics, clinic characteristics, and patient outcomes with the motorcycle accidents in order to make assumptions on whether tractor accidents should be placed in the classification of major trauma according to the ATLS system. The results of the analyses revealed that the trauma patterns, injury types and severities, and patient outcomes in tractor accidents were comparable, even worse, to motorcycle accidents. The thoracic and spinal injuries, which may be either life threatening or life-long debilitating injuries, were more frequent in tractor accidents. On the other hand, motorcycle accidents are prone to more extremity injury than tractor accidents. Another striking finding was the high rate of abdominal and pelvic injuries in tractor accidents. Although the differences in these rates between accident types were not statistically significant, there was a marginal significance that shows a clinical importance of these injuries in tractor accidents.

The motorcycle accidents are considered major trauma criteria in ATLS classifications. The mechanism of these accidents is considered as high-kinetic energy incidents, and the patients who had accidents with speeds over 50 km/h were included in this study. Despite this high-kinetic energy nature of motorcycle accidents, tractor accidents occur in lower speeds; however, the relevant injuries can be more severe due to the mechanism of trauma. Nevertheless, tractor accidents are not considered as a criterion for major trauma in ATLS system.

In general, the injuries in tractor accidents are considered to be rollover accidents. There have been significant national and international regulatory efforts in different countries to establish rollover protection structures (ROPS) on tractors to increase the safety of these vehicles. European Economic Community (EEC, now EU), Organization for Economic Cooperation and Development (OECD), International Labor Office (ILO), and International Organization for Standardization (ISO) are among the international regulatory authorities that put significant effort to bring standardization to ROPS in tractor manufacturing [9]. Without proper safety accessories on tractors, more severe crush injuries are observed. Also, poor ground and weather conditions in agricultural work, inappropriate personal safety equipment, and unsafe person transport on tractors play a role in severe injuries in these accidents [10].

The other forms of injuries in tractor accidents are falls from tractors and run overs, each of which accounts for approximately 15-20% of all tractor accidents [11, 12]. While rollovers can be observed mostly in young and adult population, drops and run overs are major mechanisms in accidents involving children [13–15]. In our study, victims of tractor accidents were older than motorcycle accident victims, and rates of female patients were higher in tractor accidents. This was partly related to high involvement rates of female agriculture-workers in the field.

The management of trauma patients is based on a multidisciplinary approach that starts with an efficient triage, which has a significant effect on the patients' outcomes. The trauma team activation criteria play a major role in the initial evaluation of these patients. Transporting patients to appropriate healthcare facilities or trauma centers may determine their fates. The classification of mortality in tractor accidents is not different from other types of accidents or injuries. Immediate deaths occur at the trauma scene due to fatal injuries in major organ systems; early deaths occur in hours following the injury and generally due to hemorrhage and cardiovascular collapse; and late injuries occur in days and weeks following the trauma and due to sepsis or multiorgan failure [16, 17]. Fast and organized triage and transportation of these patients to a critical care facility will undoubtedly determine the outcomes. However, unfortunately, many of these patients cannot reach trauma centers on time. A previous study by Newgard et al. reported that only 29.4% of critically injured patients in rural areas were initially transferred to major trauma centers, whereas this rate was 88.5% for patients in urban areas [18]. Also, authors reported that death rates within 24 hours were 89.6% in rural trauma patients and 64% in urban trauma patients. Since tractor accidents are one of the major traumas of rural areas, recognition of them as a criterion in ATLS systems will improve the outcomes of these patients due to their efficient initial triage.

### 4.1. Conclusion

To the best of our knowledge, this is the first study that evaluated the general characteristics and outcomes of tractor accidents comparatively to motorcycle accidents in Turkey. The main objective of this evaluation was to determine whether tractor accidents should be considered as a criterion in ATLS major trauma classification system. Our results revealed that the GCS and RTS scores were similar between tractor and motorcycle accidents; however, rates of major thoracic and spinal injuries were higher in tractor accidents. This finding is critical for considering tractor accidents as major traumas, which should be transported to a trauma center in initial triage.

In conclusion, the findings of this study support the hypothesis that tractor accidents should be included in the criteria of ATLS major trauma classification system and trauma team activation procedures.

## Figures and Tables

**Table 1:** General characteristics of the patients.

		Tractor	Motorcycle	P
		(n=88)	(n=339)	

*Age, year*	mean±SD median (min-max)	50.8±17.4 50.5 (17-87)	31.3±14.5 26 (18-74)	<0.001
*GCS*	mean±SD median (min-max)	14.2±2.5 15 (3-15)	14.6±1.8 15 (3-15)	0.062
*RTS*	mean±SD median (min-max)	11.6±1.6 12 (3-12)	11.8±1.3 12 (0-12)	0.201
*Duration, hour*	mean±SD median (min-max)	2.9±2.1 2 (0.8-12)	2.7±1.7 2 (0.25-12)	0.481
*Gender*	n (%)			0.001
Female		20 (22.7)	32 (9.4)	
Male		68 (77.3)	307 (90.6)	
*Route of admission*	n (%)			0.810
Emergency ambulance		71 (80.7)	281 (82.9)	
Private car		8 (9.1)	24 (7.1)	
Walking		9 (10.2)	34 (10)	

GCS, Glasgow coma score; RTS, revised trauma score.

**Table 2:** Types of traumas in the study groups.

	Tractor	Motorcycle	P
	(n=88)	(n=339)	
	n (%)	n (%)	

*Head trauma*			
Yes	43 (48.9)	135 (39.8)	0.125
No	45 (51.1)	204 (60.2)	
*Maxillofacial injury*			
Yes	16 (18.2)	59 (17.4)	0.864
No	72 (81.8)	280 (82.6)	
*Thoracic trauma*			
Yes	37 (42)	78 (23)	<0.001
No	51 (58)	261 (77)	
*Abdominal trauma*			
Yes	27 (30.7)	71 (20.9)	0.053
No	61 (69.3)	268 (79.1)	
*Pelvic injury*			
Yes	19 (21.6)	46 (13.6)	0.062
No	69 (78.4)	293 (86.4)	
*Spinal injury*			
Yes	15 (17)	20 (5.9)	0.002
No	73 (83)	319 (94.1)	
*Extremity injury*			
Yes	43 (48.9)	237 (69.9)	<0.001
No	45 (51.1)	102 (30.1)	

**Table 3:** Computerized tomography imaging findings.

	Tractor	Motorcycle	P
	(n=88)	(n=339)	
	n (%)	n (%)	

*Cranial CT*			
Normal	74 (84.1)	301 (88.8)	0.230
Linear fracture	2 (2.3)	9 (2.7)	
Compression fracture	1 (1.1)	2 (0.6)	
Subarachnoid hemorrhage	7 (8)	8 (2.4)	
Subdural hemorrhage	-	4 (1.2)	
Epidural hemorrhage	-	7 (2.1)	
Concussion	4 (4.5)	5 (1.5)	
Skull base	-	2 (0.6)	
Skin laceration	-	1 (0.3)	
*Facial CT*			
Normal	78 (88.6)	313 (92.3)	0.266
Single bone fracture	5 (5.7)	17 (5)	
Multiple bone fracture	3 (3.4)	8 (2.4)	
Other	2 (2.3)	1 (0.3)	
*Thoracic CT*			
Normal	68 (77.3)	311 (91.7)	<0.001
Pneumothorax	12 (13.6)	11 (3.2)	
Hemothorax	1 (1.1)	13 (3.8)	
Rib fracture	17 (19.3)	21 (6.2)	
Hemopneumothorax	3 (3.4)	0 (0)	
Contusion	6 (6.8)	5 (1.5)	
Flail chest	1 (1.1)	0 (0)	
Large vessel injury	-	6 (1.8)	
*Abdominal CT*			
Normal	80 (90.9)	329 (97.1)	0.011
Hepatic injury	3 (3.4)	1 (0.3)	
Splenic injury	2 (2.3)	4 (1.2)	
Hollow organ injury	1 (1.1)	3 (0.9)	
Intra-abdominal hemorrhage	1 (1.1)	1 (0.3)	
Renal injury	-	1 (0.3)	
Other	1 (1.1)	0 (0)	
*Pelvic CT*			
Normal	81 (92)	328 (96.8)	0.050
Single bone fracture	2 (2.3)	4 (1.2)	
Multiple bone fracture	4 (4.5)	7 (2.1)	
Acetabulum fracture	1 (1.1)	0 (0)	
*Spinal CT*			
Normal	75 (85.2)	327 (96.5)	<0.001
Vertebral fracture	13 (14.8)	12 (3.5)	
*Extremity CT*			
Normal	47 (53.4)	110 (32.4)	<0.001
Single long bone fracture	9 (10.2)	35 (10.3)	
Multiple long bone fracture	4 (4.5)	17 (5)	
Other fracture	4 (4.5)	21 (6.2)	
Neurovascular injury	1 (1.1)	2 (0.6)	
Muscle-tendon injury	2 (2.3)	6 (1.8)	
Superficial injury	21 (23.9)	148 (43.7)	

CT, computerized tomography.

**Table 4:** Patient outcomes.

	Tractor	Motorcycle	P
	(n=88)	(n=339)	
	n (%)	n (%)	

*Hospitalization*			0.008
No	45 (51.1)	233 (68.7)	
Inpatient clinic	29 (33)	75 (22.1)	
Intensive care unit	14 (15.9)	31 (9.1)	
*Inpatient clinics*			
Orthopedics	9 (10.2)	43 (12.7)	
General surgery	3 (3.4)	8 (2.4)	
Neurosurgery	10 (11.4)	17 (5)	
Chest surgery	7 (8)	5 (1.5)	
Other	1 (1.1)	1 (0.3)	
Plastic surgery	1 (1.1)	7 (2.1)	
Reanimation	8 (9.1)	19 (5.6)	
2^nd^ step intensive care unit	4 (4.5)	4 (1.2)	

## Data Availability

The data that support the findings of this study are available from the corresponding author upon reasonable request.
